# The relationship between resistance exercise and cardiometabolic health: a cross-sectional analysis of the 1970 British cohort study

**DOI:** 10.1007/s11332-026-01860-0

**Published:** 2026-07-28

**Authors:** Ravi K. Narang, John J. Mitchell, Mark Hamer, Joanna M. Blodgett

**Affiliations:** 1 Institute of Sport, Exercise and Health, University College London, 170 Tottenham Court Road, London, W1T 7HA UK; 2 University College London Hospitals NIHR Biomedical Research Centre, 170 Tottenham Court Road, London, UK

**Keywords:** Accelerometer, Cardiometabolic health, Moderate-vigorous physical activity, Resistance exercise

## Abstract

**Purpose:**

Resistance exercise (RE) has been shown to have a positive impact on cardiometabolic health, independent of moderate-vigorous physical activity (MVPA). However, previous studies primarily used self-reported MVPA data, which are prone to bias. The aim of this study was to examine if the association between RE frequency and cardiometabolic health was independent of device-measured MVPA.

**Methods:**

Cross-sectional analyses were conducted using participants from the 1970 British Cohort Study. This included up to 4,881 participants (age 46–48) with outcome-specific analytical samples. RE frequency data were collected via self-report. MVPA data were collected via thigh-worn accelerometry. Cardiometabolic outcomes included systolic blood pressure (SBP), total cholesterol:high-density lipoprotein cholesterol (TC:HDL-C) ratio, and HbA1c. Associations between RE frequency and each outcome were tested using multivariable linear regression and presented as percent differences. Covariates included sex, MVPA, smoking status, alcohol consumption, educational attainment, self-rated health, and disability.

**Results:**

Compared to ‘no RE’, RE frequency was inversely associated with SBP (percent difference [95% CI]; 2–4 times/month: −1.28% [−2.38, −0.19]; ≥ 2 times/week: −1.12% [−2.11, −0.14]), TC:HDL-C (2–4 times/month: −5.00% [−8.19, −1.81]; ≥ 2 times/week: −7.04% [−9.89, −4.19]), and HbA1c (2–4 times/month: -3.04% [−4.76,−1.32]; ≥ 2 times/week: -3.03% [−4.56,−1.50]) in models adjusted for sex and MVPA. These associations were attenuated after adjusting for all covariates, except for an inverse association between RE frequency ≥ 2 times/week and TC:HDL-C (−3.99% [−6.83,−1.15]).

**Conclusion:**

We identified consistent inverse associations between RE frequency and cardiometabolic outcomes. Associations were largely maintained after adjustment for sex and device-measured MVPA, but were mostly attenuated after additional adjustment for behavioral and health-related covariates. Only TC:HDL-C remained independently associated with high-frequency RE, suggesting that regular RE may be associated with a more favorable TC:HDL-C profile independent of MVPA.

**Supplementary Information:**

The online version contains supplementary material available at https://doi.org/10.1007/s11332-026-01860-0.

## Introduction

Cardiovascular disease (CVD) is a major cause of premature morbidity and mortality [1]. Cardiometabolic risk factors, such as hypertension, dyslipidemia, and diabetes mellitus, are important drivers of CVD [2]. The benefits of moderate-to-vigorous physical activity (MVPA) on these cardiometabolic risk factors have been well-reported [3–6]. In contrast, evidence for the benefits of resistance exercise (RE) on cardiometabolic health is less extensive. While RE and MVPA both involve repeated bouts of muscular contraction, RE induces greater skeletal muscle strength and hypertrophy adaptations compared to MVPA [7]. Given that skeletal muscle plays an important role in lipid [8] and glucose [9] metabolism, and that RE has positive effects on vascular and endothelial function [10], it is plausible that RE may be associated with more favorable cardiometabolic profiles independent of MVPA. Indeed, observational studies have demonstrated an association between RE participation and improved cardiometabolic outcomes, such as blood pressure (BP), lipid profile, and fasting glucose [11–17]. One challenge in separating independent effects of RE and MVPA is that these associations may partly be explained by the clustering of RE participation with other favorable health behaviors [18].

Despite numerous studies examining the favorable effects of RE independent of MVPA, these studies have relied on self-reported data to determine MVPA, which are prone to measurement errors, such as recall bias, social desirability bias, and social approval bias [19]. More recently, the use of device-based measures of movement (e.g., accelerometry) has been growing in physical activity (PA) research [20]. These devices can accurately quantify levels of PA, including duration and intensity. They can also detect small periods of ‘incidental’ PA (such as short periods of fast walking) that may not be captured in self-report measures [21] but may be important for overall health [22].

To our knowledge, no observational study has examined whether the association between RE and cardiometabolic health varies with MVPA levels. This is highly relevant, as given both MVPA and RE have beneficial effects on cardiometabolic health [3–6], it is possible that any association between RE and cardiometabolic outcomes may only be observed in those with low levels of MVPA, be attenuated in those with high levels of MVPA or in contrast, exert positive benefits on cardiometabolic health independent of MVPA levels.

Furthermore, no study has examined whether the association between RE and cardiometabolic health differs according to sex. This is an important question, as some of the mechanisms that modulate cardiometabolic health in response to RE occur within skeletal muscle [9, 23, 24], and sex-specific differences in skeletal muscle characteristics have been reported [25, 26]. In addition, sex-specific differences in skeletal muscle adaptation following a period of resistance training have also been described [27–29].

This study aims to explore the relationship between RE and key markers of cardiometabolic health. Specifically, we sought to (1) examine if associations between RE frequency and systolic blood pressure (SBP), total cholesterol:high-density lipoprotein cholesterol (TC:HDL-C), and serum hemoglobin A1c (HbA1c) were independent of device-measured MVPA, and (2) examine whether any observed associations differed according to levels of MVPA or sex. We hypothesized that RE frequency would be inversely associated with the cardiometabolic health outcomes, with these associations varying according to MVPA levels and sex.

## Methods

### Study population

**Fig. 1:**
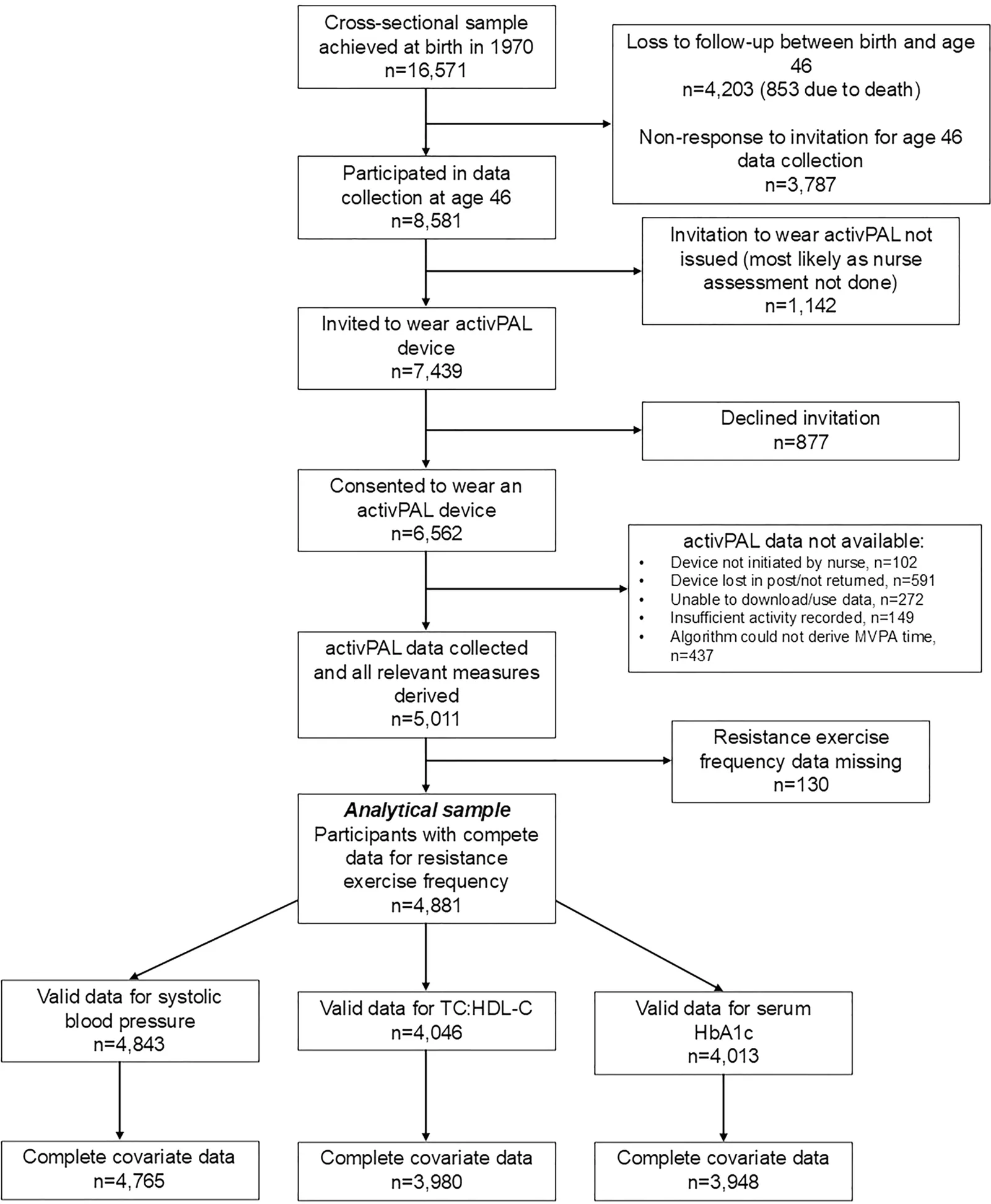
Flow chart indicating derivation of the analytical sample. *HbA1c* hemoglobin A1c, *MVPA* moderate-vigorous physical activity, *TC:HDL-C* total cholesterol:high-density lipoprotein cholesterol

This research was conducted using data from the 1970 British Cohort Study (BCS70). BCS70 is a prospective cohort study following participants from England, Scotland, and Wales, who were born in a single week in 1970 [30]. Data from the 2016–2018 sweep (participants aged 46–48) were used for this study. This sweep included biochemical measurements, questionnaires relating to demographics, health, and lifestyle, and an invitation to wear a thigh-mounted accelerometer device (Centre for Longitudinal Studies). Participants provided informed consent prior to participation and ethical approval was obtained from the National Research Ethics Committee South-East Coast—Brighton and Sussex (Ref 15/LO/1446). Participants were excluded if there was insufficient accelerometer wear-time, or missing data for RE, covariates, and outcomes. Details of study population derivation are presented in Fig. 1.

To investigate the impact of missing data, we examined differences in characteristics between the included analytical sample (participants with complete data for RE frequency) and those excluded due to missing data or loss to follow-up. This analysis demonstrated that the analytical sample was more likely to have a higher academic qualification, report a higher general state of health, was less likely to smoke, and less likely to have a disability, compared to the excluded sample (Online Resource, Table S1). In addition, the analytical sample was less likely to be on antihypertensive medication and medication for glucose regulation, but more likely to be on medication for dyslipidemia. For the outcome variables, the analytical sample had a lower median SBP, TC:HDL-C, and HbA1c. There were no between-group differences for sex, RE frequency, or MVPA.

### Measurements

Weight-based RE frequency was ascertained via self-report from the European Prospective Investigation into Cancer Study-Norfolk Physical Activity Questionnaire (EPAQ) [32]. This questionnaire has been validated against daytime energy expenditure and measures of cardiorespiratory fitness [32]. For RE, participants were asked to ‘*indicate how often you did each activity on average over the last 12 months*’. Data for the activity, ‘*Exercises with weights*’ were used for RE frequency and participants selected from the following options: ‘*none*’, ‘*less than once a month*’, ‘*once a month*’, ‘*2 to 3 times a month*’, ‘*once a week*’, ‘*2 to 3 times a week*’, ‘*4 to 5 times a week*’, or ‘*6 times a week or more’*. Due to small response rates in the higher RE frequency categories, these options were collapsed to: ‘none’, ‘ ≤ 1 time/month’ (includes ‘*less than once a month*’ and ‘*once a month*’), ‘2–4 times/month’ (includes ‘*2 to 3 times a month*’ and ‘*once a week’*) and ‘ ≥ 2 times/week’ (includes ‘*2 to 3 times a week*’, ‘*4 to 5 times a week*’, and ‘*6 times a week or more’*).

Data relating to PA were measured using a thigh-mounted accelerometer device (activPAL3 micro; PAL Technologies Ltd., Glasgow, UK). This device has been shown to be a reliable and valid tool for use in PA research [33]. The device was waterproofed and fitted on the anterior aspect of the upper thigh at the midline by a trained nurse, and participants were asked to wear the device continuously for 7 consecutive days [34]. A minimum of 1 day with ten uninterrupted hours of device wear time was required for inclusion. Data were processed using the activPAL3 software to isolate valid wear data [35]. MVPA (hours/day) was defined as a step cadence ≥ 100 [36].

### Outcomes

Cardiometabolic health outcomes that were tested included SBP, TC:HDL-C ratio, and HbA1c. A nurse performed BP measurements and nonfasting blood sampling in accordance with standard protocols (Centre for Longitudinal Studies). The TC:HDL-C ratio was calculated as this has greater predictive value for CVD compared to each component independently [37, 38]. As the use of medications may affect outcome variables and introduce measurement error, adjustments to outcome variables were made if participants were on the following classes of medication: antihypertensive medication (+ 10 mmHg for SBP [39]), drugs for dyslipidemia (+ 25% for TC, −5% for HDL-C [40]), and drugs for blood glucose regulation (+ 11 mmol/mol for HbA1c [41]).

### Covariates

Covariates were selected a priori based on known associations with RE and cardiometabolic health [42–48]. These included ‘*smoking status’* (never, ex-smoker, less than daily, daily), ‘*alcohol consumption’* (Alcohol Use Disorders Identification Test-Primary Care Scale: abstinent; irregular or regular non-risky; risky [≥ 14 units/week] [49]), ‘*educational attainment’* (left school before 16, GCSE or O level, A level or regional equivalent, diploma or degree level, higher degree), ‘*self-rated health’* (excellent, very good, good, fair, poor), and ‘*disability’* (European Statistics of Income and Living Condition classification: no long-standing health condition; to some extent; severely hampered [50]).

### Statistical analysis

As the EPAQ was not validated for RE, RE frequency data were tested for association with maximum grip strength using the Kruskal–Wallis test. RE frequency and maximum grip strength were associated in males and females (*P* < 0.05) (Online Resource, Table S2). The characteristics of participants according to sex were summarized using descriptive statistics (median and quartiles, number and percent). Comparisons between sexes for categorical variables were made using Pearson’s Chi-squared test and the Mann–Whitney U test for continuous variables. Frequency histograms for continuous variables are shown in Fig. S1 (Online Resource). The Kolmogorov–Smirnov test, along with visual inspection of the distributional shape was used to test for normality for the following variables: MVPA, SBP, TC:HDL-C, and HbA1c. Due to evidence of non-normal distributions, continuous variables were transformed to the 100log_e_ scale for subsequent analyses [51]. Due to the potential for sex-specific differences in the relationship between RE frequency and each outcome (SBP, TC:HDL-C, and HbA1c), we first tested for an interaction between RE frequency and sex for each outcome using a global interaction test. If an interaction was present, we performed subsequent analyses with data stratified by sex. This analysis was repeated to test for an interaction between RE frequency and MVPA; here MVPA was entered as an untransformed continuous variable and the overall interaction was assessed as a multi-degree of freedom test. For the main analysis, associations between RE frequency and each outcome were tested using linear regression in a model adjusted for sex, a model additionally adjusted for MVPA, and a model additionally adjusted for all remaining covariates (smoking status, alcohol consumption, educational attainment, self-rated health, and disability). Attenuation of estimates across models will be interpreted as potential confounding or behavioral clustering. To ease interpretation, coefficients from each regression analysis are presented as percent differences (sympercents) in cardiometabolic outcome of each RE frequency category compared to the ‘no RE’ category (reference group) [51]. To test the robustness of findings in the main analyses, we performed additional sensitivity analyses using raw (medication-unadjusted) outcome values. Data were reported at significance α < 0.05 and were analyzed using IBM SPSS Statistics 29 software (IBM Corp, Armonk, NY, USA). Correction for multiple testing was not applied due to concerns of Type 2 error and therefore analyses are to be interpreted as exploratory.

## Results

### Characteristics of participants

Characteristics of the study population with complete RE frequency data (*n *= 4881) are shown in Table 1, stratified by sex.

**Table 1:** Characteristics of the study population according to sex

	Male *n *= 2326	Female *n *= 2555		*P* value (Male vs Female)
*Resistance exercise frequency*				
None	1604 (69.0%)		1917 (75.0%)	< 0.01^b^
≤ 1 time/month	139 (6.0%)		140 (5.5%)	
2–4 times/month	215 (9.2%)		260 (10.2%)	
≥ 2 times/week	368 (15.8%)		238 (9.3%)	
*Smoking status, n (%)*				
Never	1149 (49.4%)	1292 (50.6%)		0.41^b^
Ex-smoker	741 (31.9%)	831 (32.5%)		
Less than daily	115 (4.9%)	117 (4.6%)		
Daily	321 (13.8%)	315 (12.3%)		
*Alcohol intake (AUDIT-PC), n (%)*				
None	194 (8.4%)	292 (11.5%)		< 0.01^b^
Irregular or regular non-risky	1425 (61.4%)	1856 (72.9%)		
Risky	700 (30.2%)	398 (15.6%)		
*Highest academic qualification, n (%)*				
No formal qualifications	677 (29.6%)	581 (23.0%)		< 0.01^b^
GCSE or equivalent	698 (30.5%)	826 (32.7%)		
A level or equivalent	123 (5.4%)	150 (5.9%)		
Undergraduate or equivalent	656 (28.7%)	836 (33.1%)		
Higher degree	133 (5.8%)	136 (5.4%)		
*General state of health (self-rated), n (%)*				
Excellent	397 (17.1%)	525 (20.5%)		0.02^b^
Very good	875 (37.6%)	936 (36.6%)		
Good	675 (29.0%)	672 (26.3%)		
Fair	289 (12.4%)	312 (12.2%)		
Poor	90 (3.9%)	110 (4.3%)		
*Disability classification (EU-SILC), n (%)*				
None	2031 (87.3%)	2113 (82.7%)		< 0.01^b^
Hampered to some extent	195 (8.4%)	302 (11.8%)		
Severely hampered	100 (4.3%)	139 (5.4%)		
Use of antihypertensive medication, n (%)	125 (5.4%)	97 (3.8%)		0.01^b^
Use of medication for dyslipidemia, n (%)	29 (1.2%)	24 (0.9%)		0.30^b^
Use of blood glucose-regulating medication, n (%)	61 (2.6%)	26 (1.0%)		< 0.01^b^
Activity time of MVPA (hr/day), median (Q1-Q3)	0.77 (0.52–1.08)	0.81 (0.57–1.09)		< 0.01^c^
Systolic blood pressure (mmHg), median (Q1-Q3)^a^	128.0 (120.3–137.3)	117.7 (109.3–128.7)		< 0.01^c^
Total cholesterol (mmol/L), median (Q1-Q3)^a^	5.50 (4.80–6.20)	5.20 (4.60–5.80)		< 0.01^c^
HDL-C (mmol/L), median (Q1-Q3)^a^	1.30 (1.10–1.60)	1.60 (1.30–1.90)		< 0.01^c^
TC:HDL-C ratio, median (Q1-Q3)^a^	4.14 (3.32–5.08)	3.15 (2.59–3.87)		< 0.01^c^
Serum HbA1c (mmol/mol), median (Q1-Q3)^a^	36.0 (34.0–38.0)	35.0 (33.0–37.0)		< 0.01^c^

*AUDIT-PC* alcohol use disorders identification test-primary care, *EU-SILC* European Statistics of Income and Living Condition, *HbA1c* hemoglobin A1c, *HDL-C* high-density lipoprotein cholesterol, *MVPA* moderate-vigorous physical activity, *Q1* 25th centile, *Q3* 75th centile, *TC* total cholesterol

^a^Adjusted for medication use.

^b^Categorical variables compared using Pearson’s Chi-squared test.

^c^Continuous (non-normal distribution) variables compared using Mann–Whitney U test.

In this sample, 1360 (27.9%) participants reported some RE engagement, with 279 (5.7%) participants performing RE ‘ ≤ 1 time/month’, 475 (9.7%) performing RE ‘2–4 times/month’, and 606 (12.4%) performing ‘RE ≥ 2 times/week’. Males typically reported higher RE engagement compared to females (31% vs 25%) while males had lower MVPA time (median [Q1-Q3] 0.77 [0.52–1.08] hr/day vs 0.81 [0.57–1.09] hr/day). Overall, 362 (7.4%) participants required outcome adjustment due to medication for BP (222 [4.5%]), dyslipidemia (53 [1.1%]), or glucose regulation (87 [1.8%]). Of these, males were more likely to be taking medication for BP (5.4% vs 3.8%) and for glucose regulation (2.6% vs 1.0%) compared to females, and had higher SBP (128.0 [120.3–137.3] mmHg vs 117.7 [109.3–128.7] mmHg), TC:HDL-C ratio (4.14 [3.32–5.08] vs 3.15 [2.59–3.87]), and HbA1c (36.0 [34.0–38.0] mmol/mol vs 35.0 [33.0–37.0] mmol/mol).

Characteristics according to RE frequency are shown in Table S3 (Online Resource). Compared to those who did not undertake RE, those reporting higher RE engagement were less likely to smoke and report a disability and more likely to drink alcohol, have a higher academic qualification, and report higher self-rated health. There were no differences between RE frequency groups for use of medication for blood pressure, dyslipidemia, or glucose regulation. Participants reporting higher RE participation had higher levels of MVPA compared to those who did not undertake RE. In addition, participants who reported higher RE participation had lower TC:HDL-C and lower serum HbA1c, while no differences were observed between RE frequency groups for SBP.

### Interaction effects ofresistance exercise with sex and MVPA

We identified no interactions between RE frequency and sex for all outcomes (*P* = 0.79 for SBP, *P* = 0.53 for TC:HDL-C, and *P* = 0.74 for HbA1c), indicating associations between RE frequency and all outcomes did not differ between males and females. Therefore, we performed the main analyses across the entire study population without stratification. Similarly, we identified no interactions between RE frequency and MVPA (*P* = 0.27 for SBP, *P* = 0.90 for TC:HDL-C, and *P* = 0.67 for HbA1c), suggesting associations between RE frequency and all outcomes did not differ across MVPA levels.

### Association of resistance exercise frequency with systolic blood pressure

**Fig. 2:**
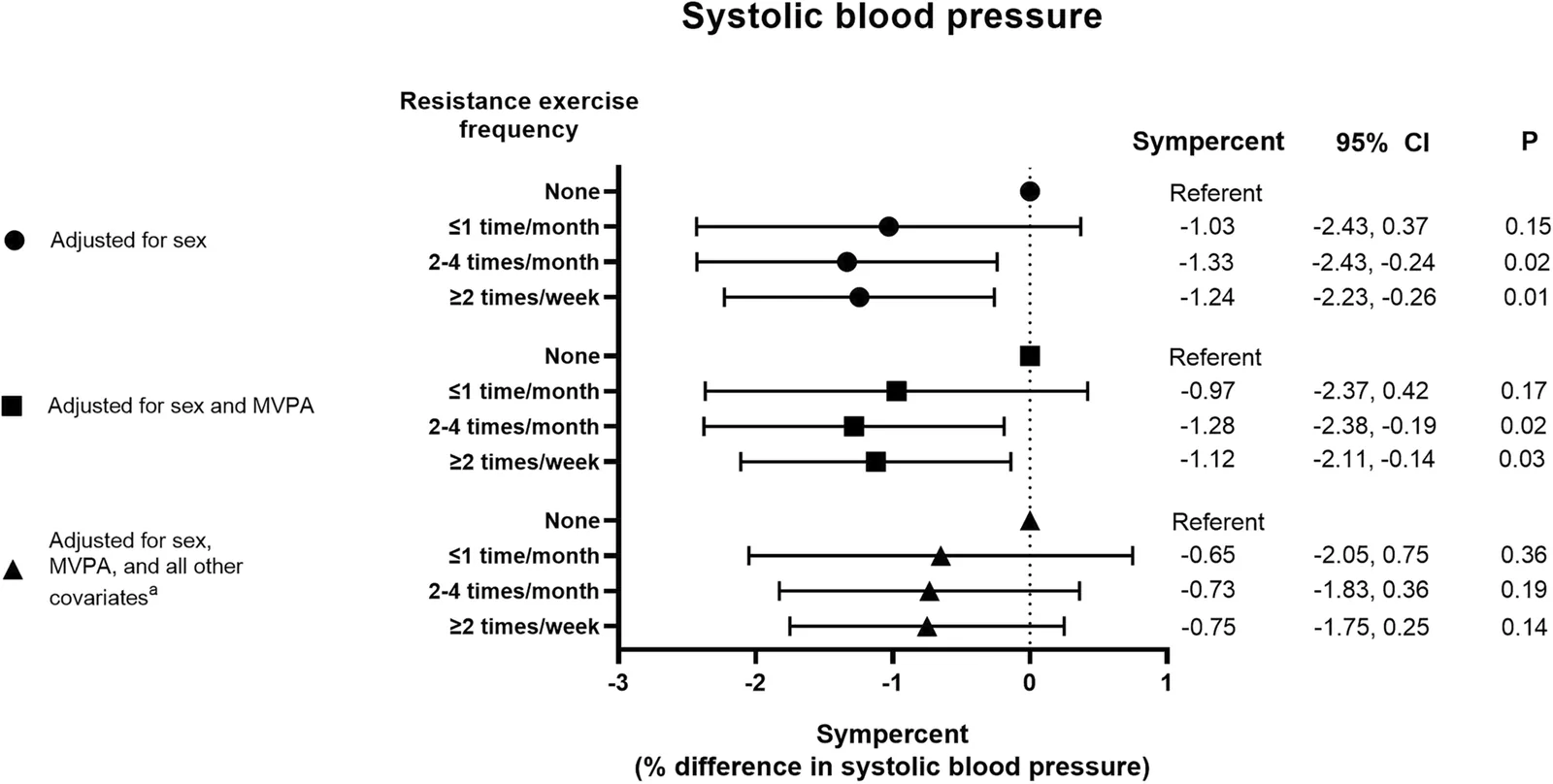
Regression analysis between resistance exercise frequency and systolic blood pressure (*n* = 4765). ^a^Other covariates include smoking status, alcohol consumption, educational attainment, self-rated health, and disability. ^b^Sympercent represents the percentage difference after transformation of the outcome variable into the 100 log_e_ scale. CI confidence interval; MVPA, moderate-vigorous physical activity

4765 participants had valid data for SBP and complete covariate data. Compared to no RE, RE ‘ ≤ 1 time/month’ was not associated with SBP (sympercent [95% CI]; −1.03% [−2.43,0.37]), while RE ‘2–4 times/month’ (−1.33% [−2.43,−0.24]) and ‘ ≥ 2 times/week’ (−1.24% [−2.23,−0.26]) were inversely associated with SBP in the sex-adjusted model (Fig. 2). We identified similar results when further adjusted for device-measured MVPA (≤ 1 time/month: −0.97% [−2.37,0.42]; 2–4 times/month: −1.28% [−2.38, −0.19]; ≥ 2 times/week: −1.12% [−2.11,−0.14]). This association was fully attenuated after adjusting for all covariates (Fig. 2, Table S4).

### Association of resistance exercise frequency with TC:HDL-C

**Fig. 3:**
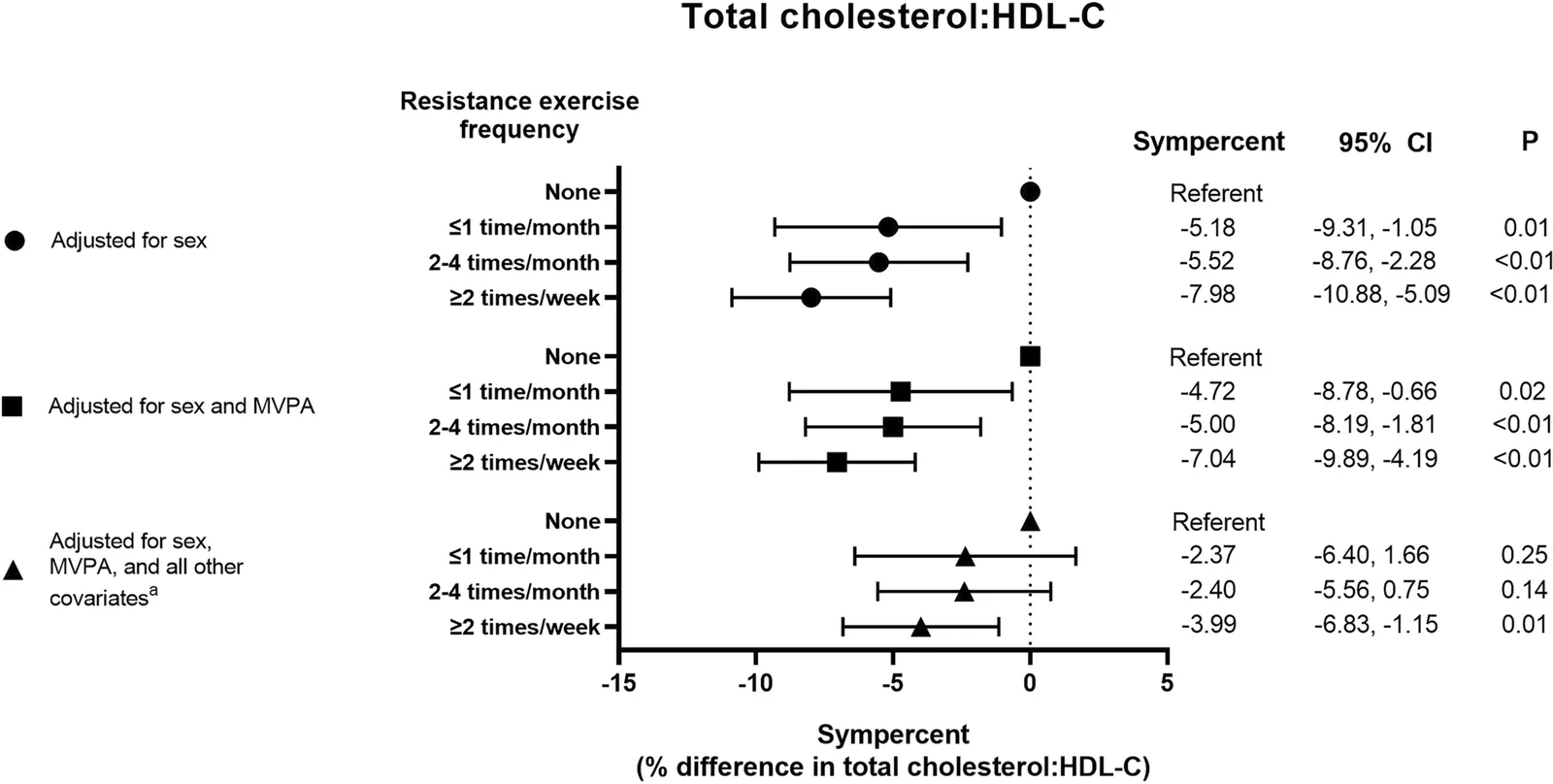
Regression analysis between resistance exercise frequency and total cholesterol:HDL-C (*n* = 3980). ^a^Other covariates include smoking status, alcohol consumption, educational attainment, self-rated health, and disability. ^b^Sympercent represents the percentage difference after transformation of the outcome variable into the 100 log_e_ scale. *CI* confidence interval, *HDL-C* high-density lipoprotein cholesterol, *MVPA* moderate-vigorous physical activity

3980 participants had valid data for TC:HDL-C and complete covariate data. Compared to no RE, an inverse association with TC:HDL-C was observed for all RE frequency categories in the sex-adjusted model (≤ 1 time/month: −5.18% [−9.31,−1.05]; 2–4 times/month: −5.52% [−8.76, −2.28]; ≥ 2 times/week: −7.98% [−10.88,−5.09]), and the model adjusted for sex and MVPA (≤ 1 time/month: −4.72% [−8.78,−0.66]; 2–4 times/month: −5.00% [−8.19, −1.81]; ≥ 2 times/week: −7.04% [−9.89, −4.19]), with the largest sympercent coefficients observed for RE ‘ ≥ 2 times/week’ (Fig. 3, Table S5). In the fully adjusted model, an inverse association was only observed for RE ‘ ≥ 2 times/week’ (−3.99% [−6.83, −1.15]).

### Association of resistance exercise frequency with HbA1c

**Fig. 4:**
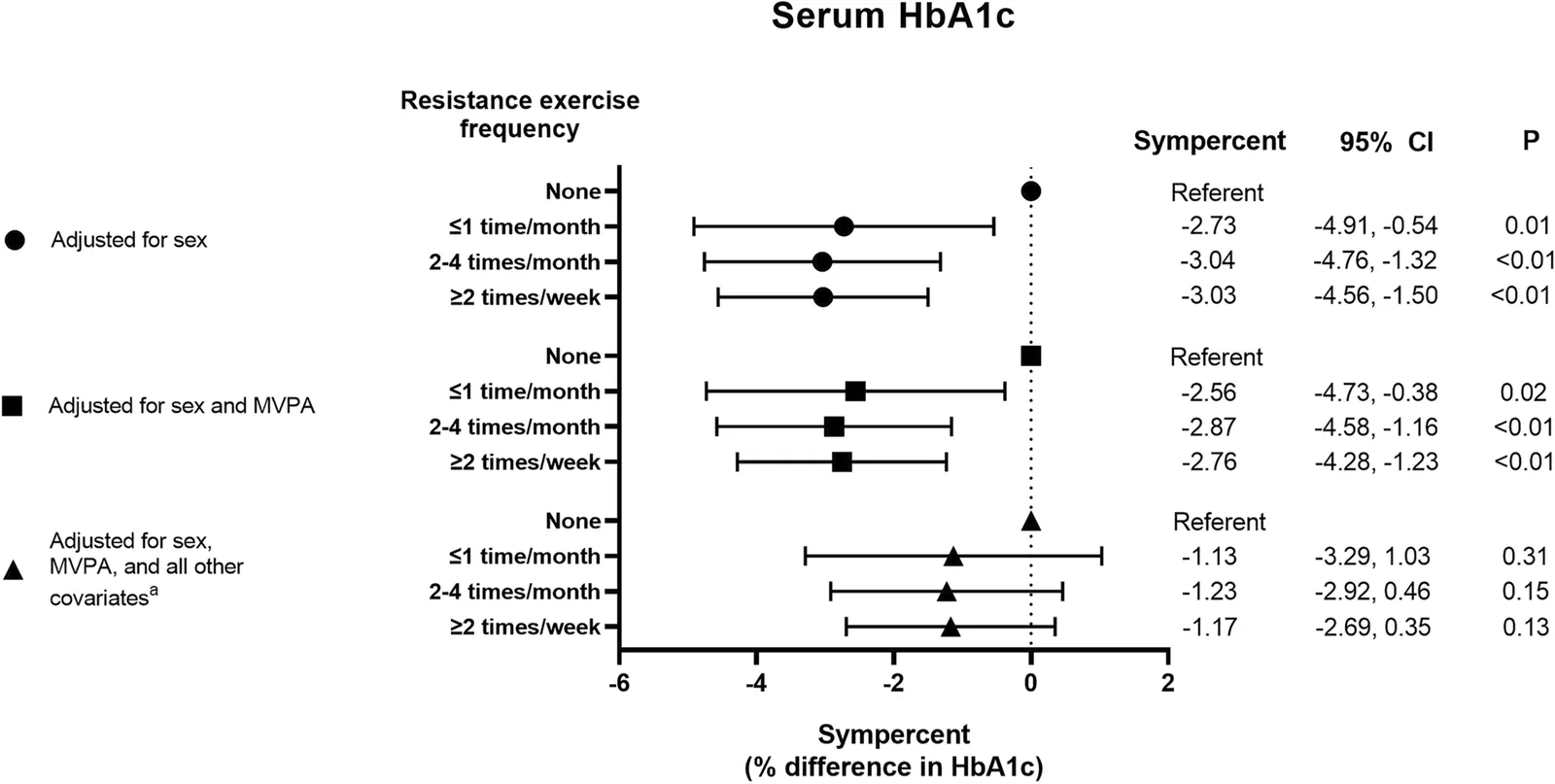
Regression analysis between resistance exercise frequency and serum HbA1c (*n* = 3948). ^a^Other covariates include smoking status, alcohol consumption, educational attainment, self-rated health, and disability. ^b^Sympercent represents the percentage difference after transformation of the outcome variable into the 100 log_e_ scale. *CI* confidence interval, *HbA1c* hemoglobin A1c, *MVPA* moderate-vigorous physical activity

3948 participants had valid data for serum HbA1c and complete covariate data. We identified an inverse association between RE frequency and HbA1c in the sex-adjusted model (≤ 1 time/month: −2.73% [−4.91, −0.54]; 2–4 times/month: −3.04% [−4.76, −1.32]; ≥ 2 times/week: −3.03% [−4.56, −1.50]) (Fig. 4, Table S6). We found similar results when further adjusting for device-measured MVPA ((≤ 1 time/month: −2.56% [−4.73, −0.38]; 2–4 times/month: −2.87% [−4.58, −1.16]; ≥ 2 times/week: −2.76% [−4.28, −1.23]). However, in the fully adjusted model, no associations were identified.

### Sensitivity analyses

Regression analyses performed without outcome adjustment for medication use were consistent with the main analyses, with similar sympercent coefficients and associations between RE frequency and each tested outcome (Online Resource, Tables S7–S9). This includes an association between RE ‘ ≥ 2 times/week’ and TC:HDL-C in a fully adjusted model. For completeness, sex-stratified regression analyses are shown in Figs. S2–S4 (Online Resource). Overall, these analyses did not substantially deviate from that of the main analysis.

## Discussion

In this study, we identified consistent inverse associations between RE frequency and key cardiometabolic health outcomes (SBP, TC:HDL-C, and HbA1c), independent of device-measured MVPA. These findings suggest that RE frequency is associated with more favorable cardiometabolic profiles after accounting for device-measured MVPA. However, except for the inverse association between RE ‘ ≥ 2 times/week’ and TC:HDL-C, all associations were fully attenuated when adjusting for health and lifestyle factors and likely reflects the clustering of health behaviors.

Our findings suggest that RE is strongly correlated with other health-related behaviors and population characteristics (e.g., smoking status, alcohol intake, education attainment, self-rated health, and disability) and may co-occur as part of a healthier behavioral profile rather than existing as an independent causal factor for the cardiometabolic outcomes. For instance, it is well-documented that lower educational attainment and poorer perceived health status are associated with less favorable cardiometabolic health [52, 53] while also associated with less RE participation [18]. For smoking status, alcohol intake, and disability, there are inconsistent associations with RE participation [18], although associations with cardiometabolic health have been reported [54, 55]. It should also be noted that the observed attenuations from our findings may have resulted from adjustment for these health-related variables on the causal pathway between resistance exercise and cardiometabolic health.

Several observational studies have previously explored the relationship between RE and cardiometabolic health, independent of self-reported MVPA [11–17]. For example, a cross-sectional analysis of > 5000 participants from the 1999–2004 National Health and Nutrition Examination Survey reported that ≥ 2 days/week of muscle-strengthening activity was associated with lower odds of pre-hypertension, dyslipidemia, and impaired fasting glucose [11]. In addition, several prospective studies have shown that RE participation was associated with a lower risk of incident hypercholesterolemia in men [12] and a lower relative risk of type 2 diabetes mellitus in men [14] and women [15]. It is important to note that most of the associations reported in these studies persisted in fully adjusted models, which included covariates such as alcohol consumption and smoking [11–17]. This contrasts with our study in which most of the associations persisted after adjustment for MVPA, but were fully attenuated when adjusting for remaining covariates. Reasons for these contrasting findings may relate to differences in the measurement of RE, covariates, and outcomes. For example, two studies assessed RE participation dichotomously [16, 17], which contrasts to the frequency measurement used in the current study; two studies used either HDL-C [11] or TC [12] as markers for dyslipidemia, both of which have been superseded by the TC:HDL-C ratio as a predictor of CVD [37, 38]; and several studies used fasting glucose [11, 17], or a diagnosis of diabetes mellitus [14, 15] as a marker of glucose dysregulation, compared to HbA1c used in the current study. Lastly, all studies measured MVPA via self-report which is prone to measurement errors [19]. There were also important differences in study samples. For example, the previous studies included a much wider age range of participants to that of the current study, with some including participants aged > 70 years [11, 14]. This is particularly relevant, as age-related differences in skeletal muscle characteristics [56] may lead to differential age-specific adaptations and cardiometabolic responses to RE. As such, further studies should explore whether associations between RE and cardiometabolic health vary according to age.

For SBP and HbA1c, there was no obvious dose–response association with RE frequency as sympercent values were similar for each RE frequency category. A possible explanation may be the presence of a ceiling effect of RE on these cardiometabolic outcomes; however, other explanations include RE frequency misclassification, inadequate data for RE training volume, or residual confounding. For TC:HDL-C, sympercent values were higher for RE frequency ‘ ≥ 2 times/week’ compared to lower RE frequency categories in all models; this suggests there may be a specific dose–response for this outcome. Churilla et al. [11] reported similar findings of a dose–response between RE frequency and dyslipidemia but no dose–response for pre-hypertension or hypertension. However, it is important to note that data for time spent undertaking RE were not available in our study, and that RE frequency data may not accurately reflect the total volume or duration of RE, which limits an accurate assessment of a dose–response.

The magnitude of association between RE frequency and each tested outcome varied, with the strongest associations observed for TC:HDL-C and the smallest seen for SBP. Although the observed associations from this study do not directly represent population-wide effects, it should be noted that small effect sizes, such as the small sympercent values observed, can theoretically have a significant impact on population-wide health. For example, a population-wide 1 mmHg reduction in SBP was associated with 20.3 and 13.3 fewer heart failure events per 1,00,000 person‐years in African Americans and whites, respectively [57], and similarly, a 1% reduction in population-wide mean TC has been shown to decrease CVD mortality by 2.5% [58]. Based on the findings of our study, any association of regular RE may be greatest for TC:HDL-C, which had sympercent reductions of up to 7% in MVPA-adjusted models.

Although mechanisms were not tested in the present study, the differences in sympercent effect sizes between outcomes may be related to the different mechanisms that modulate the effect of RE on each tested outcome. For BP, RE is thought to reduce systemic vascular resistance through a combination of increased endothelial nitric oxide-mediated vasodilation [59], increased resistance vessel endothelial function [60], and increased limb artery diameter, blood velocity, and blood flow [61]. For dyslipidemia, mechanistic evidence specific to RE is limited, but is thought to involve an increase in reverse cholesterol transport via upregulated lecithin-cholesterol acyltransferase activity and increased skeletal muscle fatty acid oxidation [23, 62]. Improvements in glucose metabolism following RE are likely due to a combination of increased skeletal muscle mass [9], upregulated skeletal muscle glucose transporter protein GLUT-4, and increased insulin sensitivity, collectively resulting in improved muscle glycogen storage and glucose utilization [24].

This study demonstrated that the relationship between RE frequency and cardiometabolic health does not vary according to levels of MVPA. Similarly, our study did not identify a differential sex-specific effect of RE on each tested outcome. However, it should be considered that characteristics such as age and ethnicity (which were relatively homogenous in our study sample), as well as previous RE experience and baseline muscle strength or mass (which were unknown for BCS70 participants), may influence any MVPA- or sex-specific differences. As such, the presence of these interaction effects should be explored in other populations.

Our study has several strengths. The use of device-measured MVPA to account for the numerous pathways linking MVPA to cardiometabolic health is superior to self-reported measures as used in prior studies given it avoids recall bias. The age-homogenous study sample eliminated age-related confounding, which is important as RE and PA behavior may vary according to age. Also, the data collection for RE allowed exploration of frequency-based patterns. However, there were limitations. The cross-sectional design means it is not possible to determine causation or rule out reverse causation. Baseline levels of physical fitness and RE training history, which may have influenced the magnitude of responses in the outcome variables, were not measured, nor were nutritional data which may be associated with exposure and outcome variables. It should be noted that adiposity measures such as body mass index and waist circumference were not included as covariates as they are likely to be on the causal pathway between RE frequency and cardiometabolic outcomes and therefore have the potential to cause overadjustment of estimates. RE data were collected via self-report which is prone to recall bias. Moreover, data collection via the survey item “Exercises with weights” did not capture the load, intensity, modality, and duration of RE which may have led to misclassification of RE and biased results towards the null. MVPA data were collected for a maximum of 7 days and may not represent participants’ usual PA behavior, particularly as PA participation has been shown to demonstrate seasonal variability [63]. For accelerometry, there may be a lack of contextual granularity whereby, if performed rapidly and dynamically, strength training could be classified as MVPA, leading to overadjustment which may obscure true associations. It is possible that covariates such as self-rated health and disability lie on the causal pathway as mediators between RE and cardiometabolic health; as such, their inclusion may have led to overadjustment, which contributed to the attenuated estimates. The analytical sample was relatively healthier compared to the excluded sample and this selection bias may have attenuated associations due to reduced variability in cardiometabolic risk within the analytical sample. Despite the use of a large sample, there was a low proportion of non-white participants, in which cardiometabolic diseases are known to differ in prevalence [64], which limits generalizability.

## Conclusion

In this cross-sectional study, we identified consistent inverse associations between RE frequency and cardiometabolic health outcomes (SBP, TC:HDL-C, and HbA1c), independent of device-measured MVPA. However, most associations were ameliorated by adjustment for behavioral and health-related factors. Only TC:HDL-C remained independently associated with high-frequency RE, suggesting that regular RE may be associated with a more favorable lipid profile independent of MVPA.

## Supplementary Information

Below is the link to the electronic supplementary material.

Supplementary file1 (DOCX 752 KB)

## Data Availability

Original study protocol and survey documents can be found online at: https://bcs70.info/ and access to these data is available through the UK data service: UK Data Service › Series. The datasets supporting this article are available in the UK Data Service repository (1970 British Cohort Study: https://beta.ukdataservice.ac.uk/datacatalogue/series/series?id=200001).
