# Supplementary material for: The relationship between resistance exercise and cardiometabolic health: a cross-sectional analysis of the 1970 British cohort study
Source: Sport Sci Health. 2026 Jul 28;22(3):282. doi: 10.1007/s11332-026-01860-0 (PMC13415302; doi:10.1007/s11332-026-01860-0)
Supplement: Supplementary file 1 — Supplementary file1 (DOCX 752 KB) [file 11332_2026_1860_MOESM1_ESM.docx]

**The Relationship Between Resistance Exercise and Cardiometabolic Health: A Cross-sectional Analysis of the 1970 British Cohort Study**

**Sports Sciences for Health**

**Supplementary Information**

**Authors**

Ravi K. Narang^a^, ravi.narang.22@ucl.ac.uk, ORCID: 0000-0002-0724-8127

John J. Mitchell^b^, john.mitchell.12@ucl.ac.uk, ORCID: 0000-0002-2719-5536

Mark Hamer^a,c^, m.hamer@ucl.ac.uk, ORCID: 0000-0002-8726-7992

Joanna M. Blodgett^a,c^, joanna.blodgett@ucl.ac.uk, ORCID: 0000-0001-7684-3571

^a^Institute of Sport, Exercise and Health, 170 Tottenham Court Road, University College London, London, W1T 7HA, UK

^b^Primary Care and Population Health, Rowland Hill Street, University College London, London, NW3 2PF, UK

^c^University College London Hospitals NIHR Biomedical Research Centre, 170 Tottenham Court Road, University College London, London, W1T 7HA, UK

**Corresponding author**

Joanna M. Blodgett, Institute of Sport, Exercise and Health, 170 Tottenham Court Road, University College London, London, W1T 7HA, UK, joanna.blodgett@ucl.ac.uk

**Table S1**

*Characteristics of the Analytical Sample and the Excluded Sample*

|  | **Analytical sample**  **n=4881** | **Excluded sample^d^**  **n=3700** | **P**  **(Included vs excluded)** |
| --- | --- | --- | --- |
| **Sex** |  | | |
| **Male** | 2326 (47.7%) | 1828 (49.4%) | **0.11^b^** |
| **Female** | 2555 (52.3%) | 1872 (50.6%) |  |
| **Resistance exercise frequency** |  | | |
| **None** | 3521 (72.1%) | 2238 (73.3%) | **0.55^b^** |
| **≤1 time/month** | 279 (5.7%) | 156 (5.1%) |  |
| **2-4 times/month** | 475 (9.7%) | 282 (9.2%) |  |
| **≥2 times/week** | 606 (12.4%) | 377 (12.3%) |  |
| **Smoking status, n (%)** |  | | |
| **Never** | 2441 (50.0%) | 1637 (44.3%) | **<0.01^b^** |
| **Ex-smoker** | 1572 (32.2%) | 1150 (31.1%) |  |
| **Less than daily** | 232 (4.8%) | 217 (5.9%) |  |
| **Daily** | 636 (13.0%) | 693 (18.7%) |  |
| **Alcohol intake (AUDIT-PC), n (%)** |  | | |
| **None** | 486 (10.0%) | 498 (13.7%) | **<0.01^b^** |
| **Irregular or regular non-risky** | 3281 (67.4%) | 2247 (61.8%) |  |
| **Risky** | 1098 (22.6%) | 888 (24.4%) |  |
| **Highest academic qualification, n (%)** |  | | |
| **No formal qualifications** | 1258 (26.1%) | 1181 (32.6%) | **<0.01^b^** |
| **GCSE or equivalent** | 1524 (31.6%) | 1136 (31.3%) |  |
| **A level or equivalent** | 273 (5.7%) | 189 (5.2%) |  |
| **Undergraduate or equivalent** | 1492 (31.0%) | 949 (26.2%) |  |
| **Higher degree** | 269 (5.6%) | 173 (4.8%) |  |
| **General state of health (self-rated), n (%)** |  | | |
| **Excellent** | 922 (18.9%) | 549 (14.9%) | **<0.01^b^** |
| **Very good** | 1811 (37.1%) | 1178 (31.9%) |  |
| **Good** | 1347 (27.6%) | 1113 (30.1%) |  |
| **Fair** | 601 (12.3%) | 566 (15.3%) |  |
| **Poor** | 200 (4.1%) | 289 (7.8%) |  |
| **Disability classification (EU-SILC), n (%)** |  | | |
| **None** | 4144 (84.9%) | 2923 (79.1%) | **<0.01^b^** |
| **Hampered to some extent** | 497 (10.2%) | 438 (11.9%) |  |
| **Severely hampered** | 239 (4.9%) | 333 (9.0%) |  |
| **Use of anti-hypertensive medication, n (%)** | 222 (4.5%) | 806 (8.3%) | **<0.01^b^** |
| **Use of medication for dyslipidemia, n (%)** | 53 (1.1%) | 24 (0.2%) | **<0.01^b^** |
| **Use of blood glucose-regulating medication, n (%)** | 87 (1.8%) | 329 (3.4%) | **<0.01^b^** |
| **Activity time of MVPA (hr/day), median (Q1-Q3)** | 0.79 (0.55-1.08) | 0.85 (0.56-1.13) | **0.42^c^** |
| **Systolic blood pressure (mmHg), median (Q1-Q3)^a^** | 123.0 (114.0-133.7) | 124.3 (114.7-135.7) | **<0.01^c^** |
| **Total cholesterol (mmol/L), median (Q1-Q3)^a^** | 5.38 (4.70-6.00) | 5.40 (4.70-6.00) | **0.69^c^** |
| **HDL-C (mmol/L), median (Q1-Q3)^a^** | 1.50 (1.20-1.80) | 1.40 (1.20-1.70) | **<0.01^c^** |
| **TC:HDL-C ratio, median (Q1-Q3)^a^** | 3.56 (2.85-4.55) | 3.71 (2.90-4.69) | **0.01^c^** |
| **Serum HbA1c (mmol/mol), median (Q1-Q3)^a^** | 36.0 (33.0-38.0) | 36.0 (34.0-39.0) | **<0.01^c^** |

^a^Adjusted for medication use.

^b^Categorical variables compared using Pearson’s chi-squared test.

^c^Continuous (non-normal distribution) variables compared using Mann-Whitney U test.

^d^Excluded sample includes individuals who did participate in data collection at the age 46 sweep, but who had missing data for resistance exercise, covariates, or outcomes.

AUDIT-PC, Alcohol use disorders identification test – primary care; EU-SILC, European Statistics of Income and Living Condition; HbA1c, haemoglobin A1c; HDL-C, high-density lipoprotein cholesterol; MVPA, moderate-vigorous physical activity; TC, total cholesterol.

**Table S2**

*Maximum grip strength according to resistance exercise frequency*

| **Resistance exercise frequency** | **Maximum grip strength (kg)** | | | | | |
| --- | --- | --- | --- | --- | --- | --- |
|  | **Male** | | | **Female** | | |
|  | **n** | **Mean (SD)** | **Median (Q1,Q3)** | **n** | **Mean (SD)** | **Median (Q1,Q3)** |
| None | 1604 | 47.9 (9.1) | 48.0 (42.0, 54.0) | 1917 | 29.7 (5.7) | 30.0 (26.0-34.0) |
| ≤1 time/month | 139 | 48.6 (7.7) | 49.0 (44.0, 53.0) | 140 | 30.3 (4.9) | 30.0 (27.0, 34.0) |
| 2-4 times/month | 215 | 48.2 (8.6) | 49.0 (43.0, 54.0) | 260 | 30.2 (5.3) | 30.0 (26.0, 33.0) |
| ≥2 times/week | 368 | 49.4 (8.0) | 50.0 (45.0, 55.0) | 238 | 30.9 (5.5) | 31.0 (28.0, 35.0) |

SD, standard deviation

**Table S3**

*Characteristics of the Study Population According to Resistance Exercise frequency*

|  | **None** | **≤1 time/month** | **2-4 times/month** | **≥2 times/week** | **P** |  |
| --- | --- | --- | --- | --- | --- | --- |
| **Sex** |  | | | | | |
| Male | 1604 (45.6%) | 139 (49.8%) | 215 (45.3%) | 368 (60.7%) | **<0.01**^b^ |  |
| Female | 1917 (54.4%) | 140 (50.2%) | 260 (54.7%) | 238 (39.3%) |  |  |
| **Smoking status, n (%)** |  | | | | | |
| Never | 1709 (48.5%) | 153 (54.8%) | 260 (54.7%) | 319 (52.6%) | **<0.01^b^** |  |
| Ex-smoker | 1092 (31.0%) | 90 (32.3%) | 172 (36.2%) | 218 (36.0%) |  |  |
| Less than daily | 174 (4.9%) | 14 (5.0%) | 16 (3.4%) | 28 (4.6%) |  |  |
| Daily | 546 (15.5%) | 22 (7.9%) | 27 (5.7%) | 41 (6.8%) |  |  |
| **Alcohol intake (AUDIT-PC), n (%)** |  | | | | | |
| None | 391 (11.1%) | 20 (7.2%) | 33 (7.0%) | 42 (6.9%) | **<0.01^b^** |  |
| Irregular or regular non-risky | 2343 (66.8%) | 188 (67.9%) | 335 (70.7%) | 415 (68.6%) |  |  |
| Risky | 775 (22.1%) | 69 (24.9%) | 106 (22.4%) | 148 (24.5%) |  |  |
| **Highest academic qualification, n (%)** |  | | | | |  |
| No formal qualifications | 968 (27.8%) | 50 (18.3%) | 84 (17.8%) | 156 (26.3%) | **<0.01^b^** |  |
| GCSE or equivalent | 1135 (32.6%) | 75 (27.5%) | 131 (27.8%) | 183 (30.9%) |  |  |
| A level or equivalent | 196 (5.6%) | 15 (5.5%) | 35 (7.4%) | 27 (4.6%) |  |  |
| Undergraduate or equivalent | 1015 (29.2%) | 109 (39.9%) | 179 (37.9%) | 189 (31.9%) |  |  |
| Higher degree | 164 (4.7%) | 24 (8.8%) | 43 (9.1%) | 38 (6.4%) |  |  |
| **General state of health (self-rated), n (%)** |  | | | | |  |
| Excellent | 576 (16.4%) | 62 (22.2%) | 103 (21.7%) | 181 (29.9%) | **<0.01^b^** |  |
| Very good | 1247 (35.4%) | 100 (35.8%) | 216 (45.5%) | 248 (40.9%) |  |  |
| Good | 1017 (28.9%) | 82 (29.4%) | 124 (26.1%) | 124 (20.5%) |  |  |
| Fair | 501 (14.2%) | 32 (11.5%) | 28 (5.9%) | 40 (6.6%) |  |  |
| Poor | 180 (5.1%) | 3 (1.1%) | 4 (0.8%) | 13 (2.1%) |  |  |
| **Disability classification (EU-SILC), n (%)** |  | | | | |  |
| None | 2943 (83.6%) | 240 (86.3%) | 421 (88.6%) | 540 (89.1%) | **<0.01^b^** |  |
| Hampered to some extent | 385 (10.9%) | 34 (12.2%) | 37 (7.8%) | 41 (6.8%) |  |  |
| Severely hampered | 193 (5.5%) | 4 (1.4%) | 17 (3.6%) | 25 (4.1%) |  |  |
| **Use of anti-hypertensive medication, n (%)** | 172 (4.9%) | 12 (4.3%) | 18 (3.8%) | 20 (3.3%) | **0.29^b^** |  |
| **Use of medication for dyslipidemia, n (%)** | 42 (1.2%) | 1 (0.4%) | 2 (0.4%) | 8 (1.3%) | **0.26^b^** |  |
| **Use of blood glucose-regulating medication, n (%)** | 73 (2.1%) | 4 (1.4%) | 4 (0.8%) | 6 (1.0%) | **0.09^b^** |  |
| **Activity time of MVPA (hr/day), median (Q1-Q3)** | 0.77 (0.53-1.07) | 0.79 (0.58-1.11) | 0.81 (0.58-1.09) | 0.85 (0.60-1.16) | **<0.01^c^** |  |
| **Systolic blood pressure (mmHg), median (Q1-Q3)^a^** | 123.3 (114.3-134.0) | 122.0 (113.3-132.3) | 122.0 (112.3-132) | 123.0 (113.7-134.0) | **0.17^c^** |  |
| **Total cholesterol (mmol/L), median (Q1-Q3)^a^** | 5.38 (4.70-6.00) | 5.40 (4.80-5.90) | 5.30 (4.80-6.00) | 5.34 (4.70-6.00) | **<0.01** |  |
| **HDL-C (mmol/L), median (Q1-Q3)^a^** | 1.50 (1.20-1.80) | 1.50 (1.20-1.80) | 1.50 (1.30-1.80) | 1.50 (1.30-1.80) | **0.99^c^** |  |
| **TC:HDL-C ratio, median (Q1-Q3)^a^** | 3.62 (2.89-4.60) | 3.51 (2.78-4.50) | 3.43 (2.79-4.42) | 3.42 (2.78-4.45) | **<0.01^c^** |  |
| **Serum HbA1c (mmol/mol), median (Q1-Q3)^a^** | 36.0 (34.0-38.0) | 35.0 (33.0-37.0) | 35.0 (33.0-37.0) | 35.0 (33.0-37.0) | **<0.01^c^** |  |

^a^Adjusted for medication use.

^b^Categorical variables compared using Pearson’s chi-squared test.

^c^Continuous (non-normal distribution) variables compared using Kruskal-Wallis test.

AUDIT-PC, Alcohol use disorders identification test – primary care; EU-SILC, European Statistics of Income and Living Condition; HbA1c, haemoglobin A1c; HDL-C, high-density lipoprotein cholesterol; MVPA, moderate-vigorous physical activity; Q1, 25^th^ centile; Q3, 75^th^ centile; TC, total cholesterol.

**Table S4**

*Linear Regression Analysis of Resistance Exercise Frequency with Systolic Blood Pressure*

| **Model**  **N=4,765** | **Resistance exercise frequency** | **Systolic blood pressure** | | | |
| --- | --- | --- | --- | --- | --- |
|  |  | **Coefficient (Sympercent)** | **95% CI** | **P** |  |
| Adjusted for sex | None | Referent | | | |
|  | ≤1 time/month | -1.03 | -2.43, 0.37 | 0.15 |  |
|  | 2-4 times/month | -1.33 | -2.43, -0.24 | 0.02 |  |
|  | ≥2 times/week | -1.24 | -2.23, -0.26 | 0.01 |  |
| Adjusted for sex and MVPA | None | Referent | | | |
|  | ≤1 time/month | -0.97 | -2.37, 0.42 | 0.17 |  |
|  | 2-4 times/month | -1.28 | -2.38, -0.19 | 0.02 |  |
|  | ≥2 times/week | -1.12 | -2.11, -0.14 | 0.03 |  |
| Adjusted for sex, MVPA, and all other covariates^a^ | None | Referent | | | |
|  | ≤1 time/month | -0.65 | -2.05, 0.75 | 0.36 |  |
|  | 2-4 times/month | -0.73 | -1.83, 0.36 | 0.19 |  |
|  | ≥2 times/week | -0.75 | -1.75, 0.25 | 0.14 |  |

^a^Other covariates include smoking status, alcohol consumption, educational attainment, self-rated health, and disability.

CI, confidence interval; MVPA, moderate-vigorous physical activity.

**Table S5**

*Linear Regression Analysis of Resistance Exercise Frequency with Total Cholesterol:HDL-C cholesterol*

| **Model**  **N=3,980** | **Resistance exercise frequency** | **Total cholesterol:HDL-C cholesterol** | | | |
| --- | --- | --- | --- | --- | --- |
|  |  | **Coefficient (Sympercent)** | **95% CI** | **P** |  |
| Adjusted for sex | None | Referent | | | |
|  | ≤1 time/month | -5.18 | -9.31, -1.05 | 0.01 |  |
|  | 2-4 times/month | -5.52 | -8.76, -2.28 | <0.01 |  |
|  | ≥2 times/week | -7.98 | -10.88, -5.09 | <0.01 |  |
| Adjusted for sex and MVPA | None | Referent | | | |
|  | ≤1 time/month | -4.72 | -8.78, -0.66 | 0.02 |  |
|  | 2-4 times/month | -5.00 | -8.19, -1.81 | <0.01 |  |
|  | ≥2 times/week | -7.04 | -9.89, -4.19 | <0.01 |  |
| Adjusted for sex, MVPA, and all other covariates^a^ | None | Referent | | | |
|  | ≤1 time/month | -2.37 | -6.40, 1.66 | 0.25 |  |
|  | 2-4 times/month | -2.40 | -5.56, 0.75 | 0.14 |  |
|  | ≥2 times/week | -3.99 | -6.83, -1.15 | 0.01 |  |

^a^Other covariates include smoking status, alcohol consumption, educational attainment, self-rated health, and disability.

CI, confidence interval; HDL-C, high-density lipoprotein cholesterol; MVPA, moderate-vigorous physical activity.

**Table S6**

*Linear Regression Analysis of Resistance Exercise Frequency with Serum HbA1c*

| **Model**  **N=3,948** | **Resistance exercise frequency** | **Serum HbA1c** | | | |
| --- | --- | --- | --- | --- | --- |
|  |  | **Coefficient (Sympercent)** | **95% CI** | **P** |  |
| Adjusted for sex | None | Referent | | | |
|  | ≤1 time/month | -2.73 | -4.91, -0.54 | 0.01 |  |
|  | 2-4 times/month | -3.04 | -4.76, -1.32 | <0.01 |  |
|  | ≥2 times/week | -3.03 | -4.56, -1.50 | <0.01 |  |
| Adjusted for sex and MVPA | None | Referent | | | |
|  | ≤1 time/month | -2.56 | -4.73, -0.38 | 0.02 |  |
|  | 2-4 times/month | -2.87 | -4.58, -1.16 | <0.01 |  |
|  | ≥2 times/week | -2.76 | -4.28, -1.23 | <0.01 |  |
| Adjusted for sex, MVPA, and all other covariates^a^ | None | Referent | | | |
|  | ≤1 time/month | -1.13 | -3.29, 1.03 | 0.31 |  |
|  | 2-4 times/month | -1.23 | -2.92, 0.46 | 0.15 |  |
|  | ≥2 times/week | -1.17 | -2.69, 0.35 | 0.13 |  |

^a^Other covariates include smoking status, alcohol consumption, educational attainment, self-rated health, and disability.

CI, confidence interval; HbA1c, glycated haemoglobin; MVPA, moderate-vigorous physical activity.

**Table S7**

*Linear Regression Analysis of Resistance Exercise Frequency with Systolic Blood Pressure – Outcome Values Unadjusted for Medication Use*

| **Model** | **Resistance exercise frequency** | **Systolic blood pressure** | | | |
| --- | --- | --- | --- | --- | --- |
|  |  | **Coefficient^b^ (Sympercent)** | **95% CI** | **P** |  |
| Adjusted for sex | None | Referent | | | |
|  | ≤1 time/month | -0.97 | -2.34, 0.40 | 0.17 |  |
|  | 2-4 times/month | -1.24 | -2.31, -0.17 | 0.02 |  |
|  | ≥2 times/week | -1.11 | -2.08, -0.14 | 0.02 |  |
| Adjusted for sex and MVPA | None | Referent | | | |
|  | ≤1 time/month | -0.92 | -2.28, 0.45 | 0.19 |  |
|  | 2-4 times/month | -1.20 | -2.27, -0.13 | 0.03 |  |
|  | ≥2 times/week | -1.01 | -1.97, -0.04 | 0.04 |  |
| Adjusted for sex, MVPA, and all other covariates^a^ | None | Referent | | | |
|  | ≤1 time/month | -0.63 | -2.00, 0.75 | 0.37 |  |
|  | 2-4 times/month | -0.70 | -1.77, 0.37 | 0.20 |  |
|  | ≥2 times/week | -0.69 | -1.66, 0.29 | 0.17 |  |

^a^Other covariates include smoking status, alcohol consumption, educational attainment, self-rated health, and disability

^b^Sympercent represents the percentage difference after transformation of the outcome variable into the 100 log_e_ scale

CI, confidence interval; MVPA, moderate-vigorous physical activity.

**Table S8**

*Linear Regression Analysis of Resistance Exercise Frequency with Total Cholesterol:HDL-C cholesterol – Outcome Values Unadjusted for Medication Use*

| **Model** | **Resistance exercise frequency** | **Total cholesterol:HDL-C cholesterol** | | | |
| --- | --- | --- | --- | --- | --- |
|  |  | **Coefficient^b^ (Sympercent)** | **95% CI** | **P** |  |
| Adjusted for sex | None | Referent | | | |
|  | ≤1 time/month | -4.91 | -9.02, -0.79 | 0.02 |  |
|  | 2-4 times/month | -5.27 | -8.50, -2.03 | <0.01 |  |
|  | ≥2 times/week | -8.00 | -10.87, -5.12 | <0.01 |  |
| Adjusted for sex and MVPA | None | Referent | | | |
|  | ≤1 time/month | -4.46 | -8.51, -0.41 | 0.03 |  |
|  | 2-4 times/month | -4.76 | -7.94, -1.58 | <0.01 |  |
|  | ≥2 times/week | -7.09 | -9.92, -4.25 | <0.01 |  |
| Adjusted for sex, MVPA, and all other covariates^a^ | None | Referent | | | |
|  | ≤1 time/month | -2.14 | -6.15, 1.88 | 0.30 |  |
|  | 2-4 times/month | -2.19 | -5.34, 0.96 | 0.17 |  |
|  | ≥2 times/week | -4.03 | -6.86, -1.20 | 0.01 |  |

^a^Other covariates include smoking status, alcohol consumption, educational attainment, self-rated health, and disability

^b^Sympercent represents the percentage difference after transformation of the outcome variable into the 100 log_e_ scale

CI, confidence interval; HDL-C, high-density lipoprotein cholesterol; MVPA, moderate-vigorous physical activity.

**Table S9**

*Linear Regression Analysis of Resistance Exercise Frequency with Serum HbA1c – Outcome Values Unadjusted for Medication Use*

| **Model** | **Resistance exercise frequency** | **Serum HbA1c** | | | |
| --- | --- | --- | --- | --- | --- |
|  |  | **Coefficient^b^ (Sympercent)** | **95% CI** | **P** |  |
| Adjusted for sex | None | Referent | | | |
|  | ≤1 time/month | -2.45 | -4.50, -0.40 | 0.02 |  |
|  | 2-4 times/month | -2.83 | -4.44, -1.22 | <0.01 |  |
|  | ≥2 times/week | -2.81 | -4.25, -1.38 | <0.01 |  |
| Adjusted for sex and MVPA | None | Referent | | | |
|  | ≤1 time/month | -2.29 | -4.33, -0.25 | 0.03 |  |
|  | 2-4 times/month | -2.67 | -4.27, -1.06 | <0.01 |  |
|  | ≥2 times/week | -2.56 | -3.99, -1.13 | <0.01 |  |
| Adjusted for sex, MVPA, and all other covariates^a^ | None | Referent | | | |
|  | ≤1 time/month | -0.94 | -2.97, 1.09 | 0.36 |  |
|  | 2-4 times/month | -1.14 | -2.73, 0.44 | 0.16 |  |
|  | ≥2 times/week | -1.08 | -2.51, 0.34 | 0.14 |  |

^a^Other covariates include smoking status, alcohol consumption, educational attainment, self-rated health, and disability

^b^Sympercent represents the percentage difference after transformation of the outcome variable into the 100 log_e_ scale

CI, confidence interval; HbA1c, glycated haemoglobin; MVPA, moderate-vigorous physical activity.

**Figure S1**

*Frequency Histograms for (A) MVPA, (B) Systolic blood pressure, (C) TC:HDL-C, and (D) HbA1c*

**
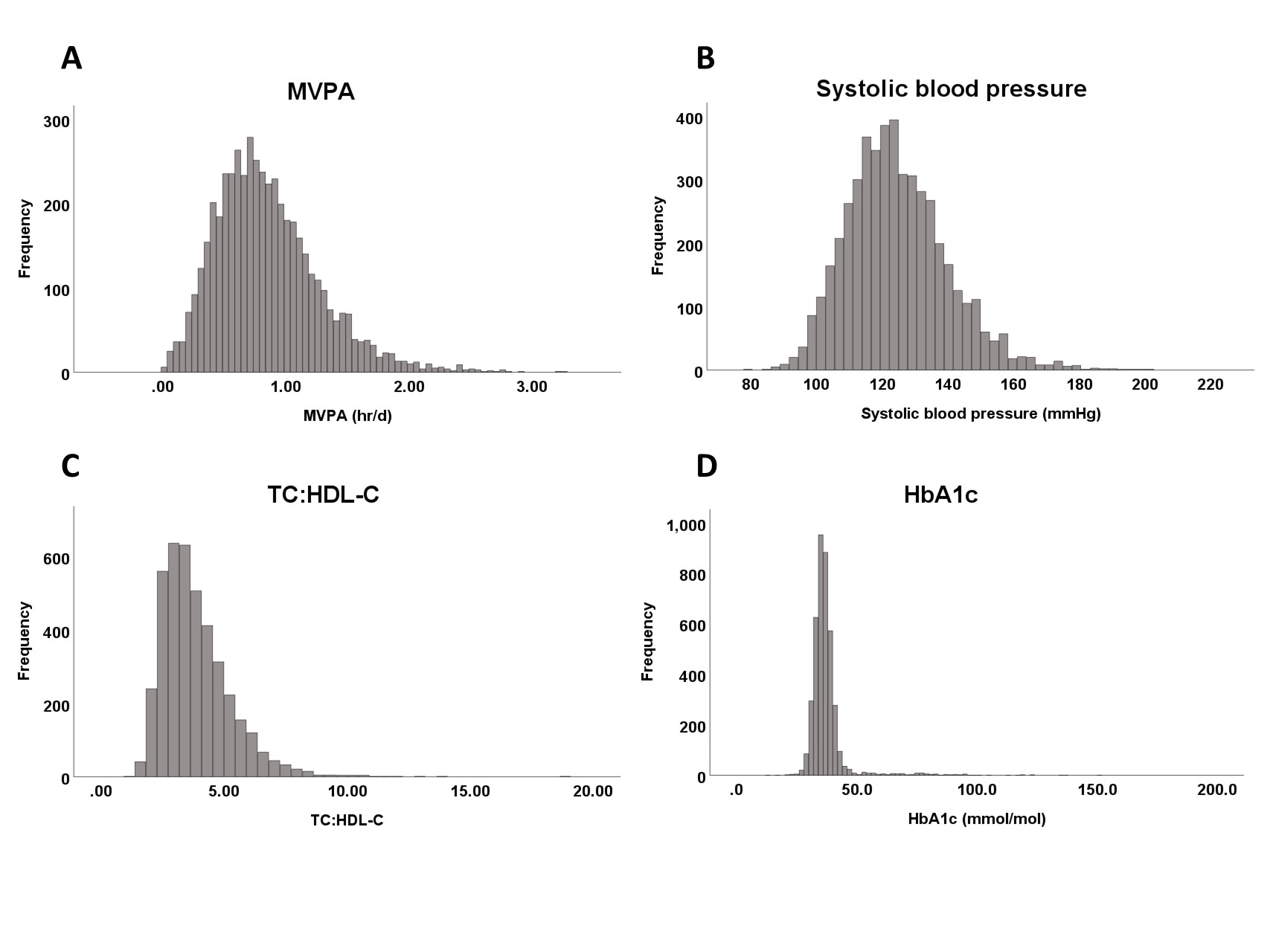
**

HbA1c, haemoglobin A1c; MVPA, moderate-vigorous physical activity; TC:HDL-C, total cholesterol:high-density lipoprotein-cholesterol.

**Figure S2**

*Regression Analysis Between Resistance Exercise Frequency and Systolic Blood Pressure – Stratified by Sex*


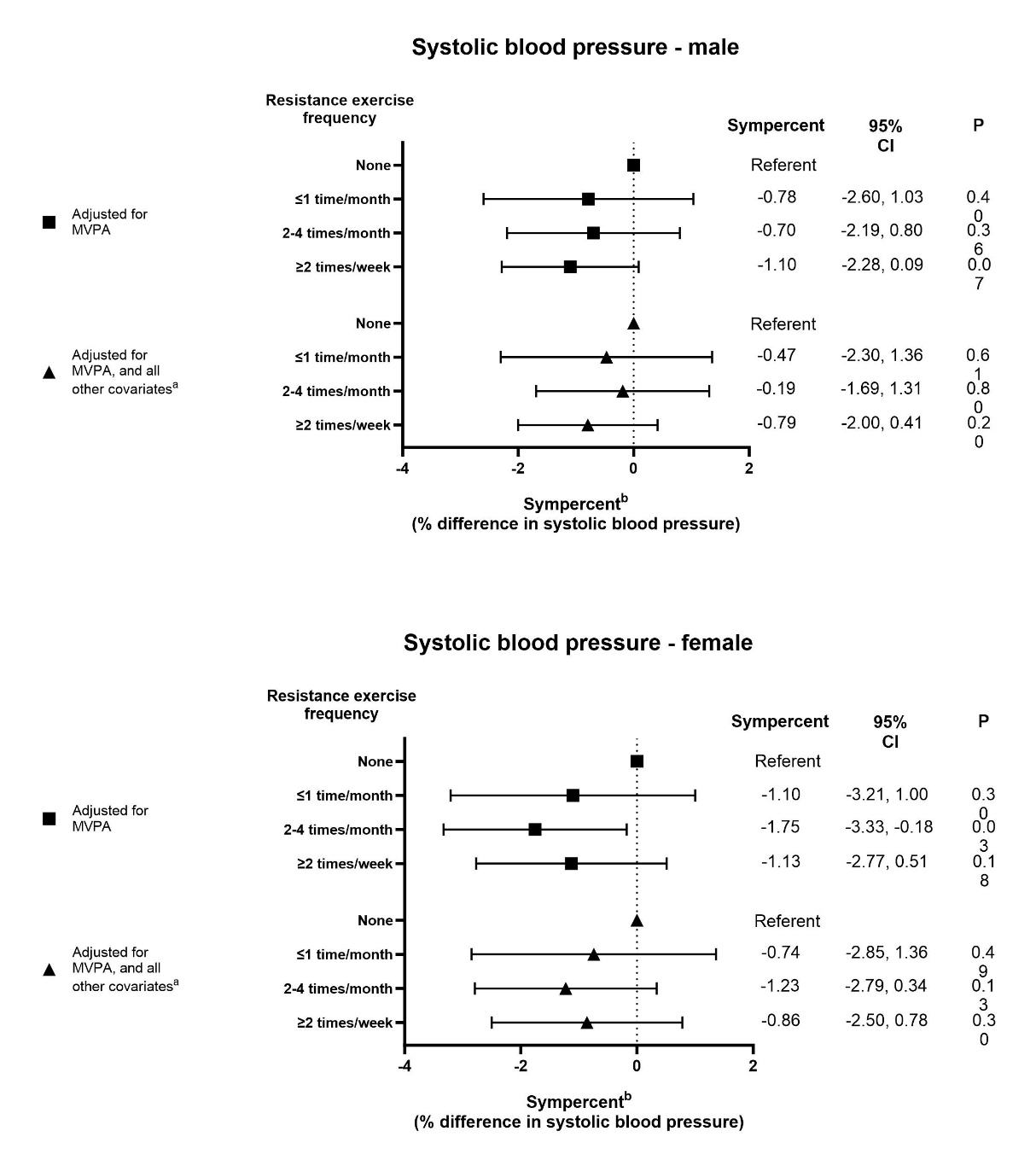


^a^Other covariates include smoking status, alcohol consumption, educational attainment, self-rated health, and disability

^b^Sympercent represents the percentage difference after transformation of the outcome variable into the 100 log_e_ scale

CI, confidence interval; MVPA, moderate-vigorous physical activity.

**Figure S3**

*Regression Analysis Between Resistance Exercise Frequency and Total Cholesterol:HDL-C – Stratified by Sex*


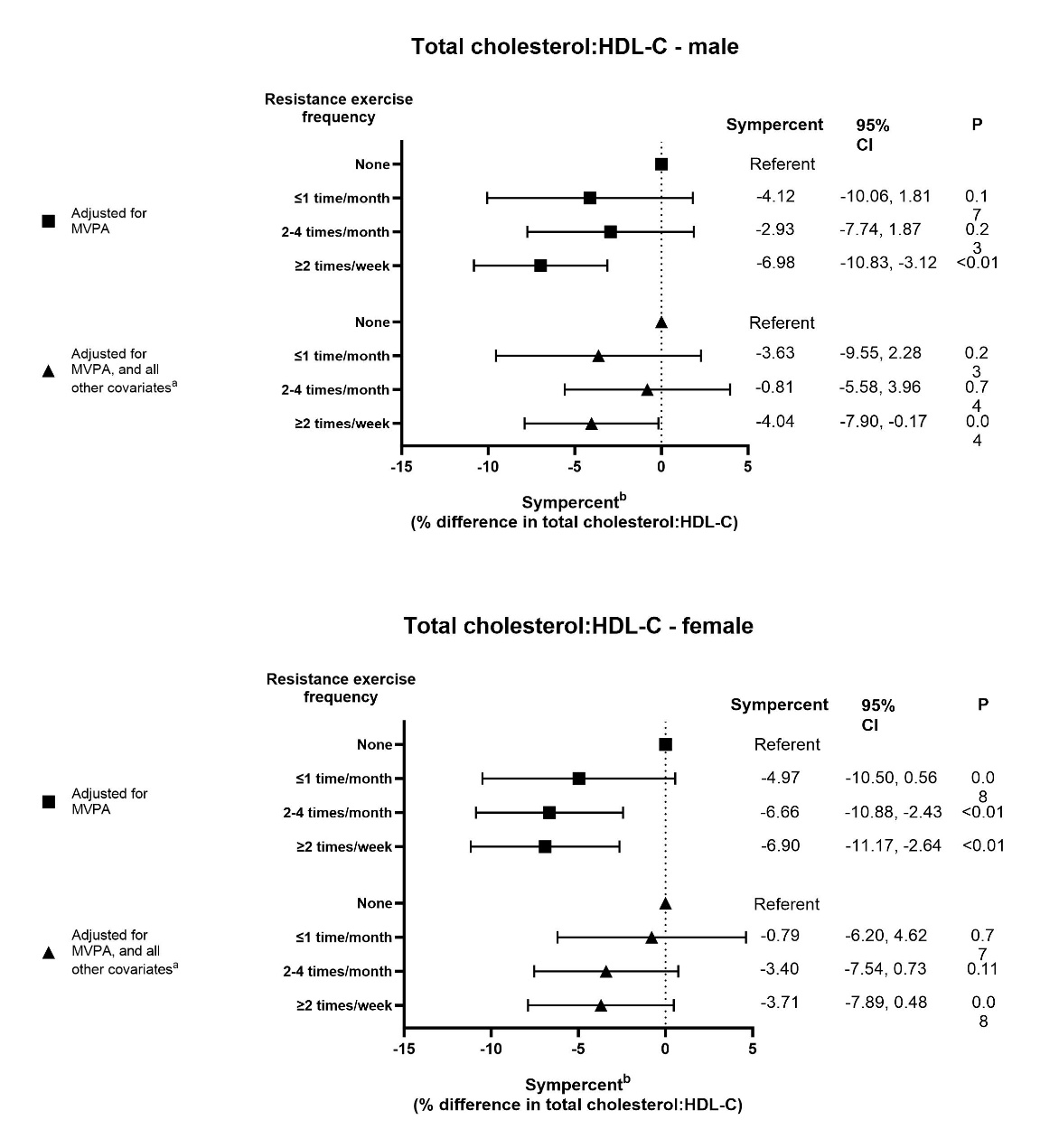


^a^Other covariates include smoking status, alcohol consumption, educational attainment, self-rated health, and disability

^b^Sympercent represents the percentage difference after transformation of the outcome variable into the 100 log_e_ scale

CI, confidence interval; HDL-C, high-density lipoprotein cholesterol; MVPA, moderate-vigorous physical activity.

**Figure S4**

*Regression Analysis Between Resistance Exercise Frequency and Serum HbA1c – Stratified by Sex*


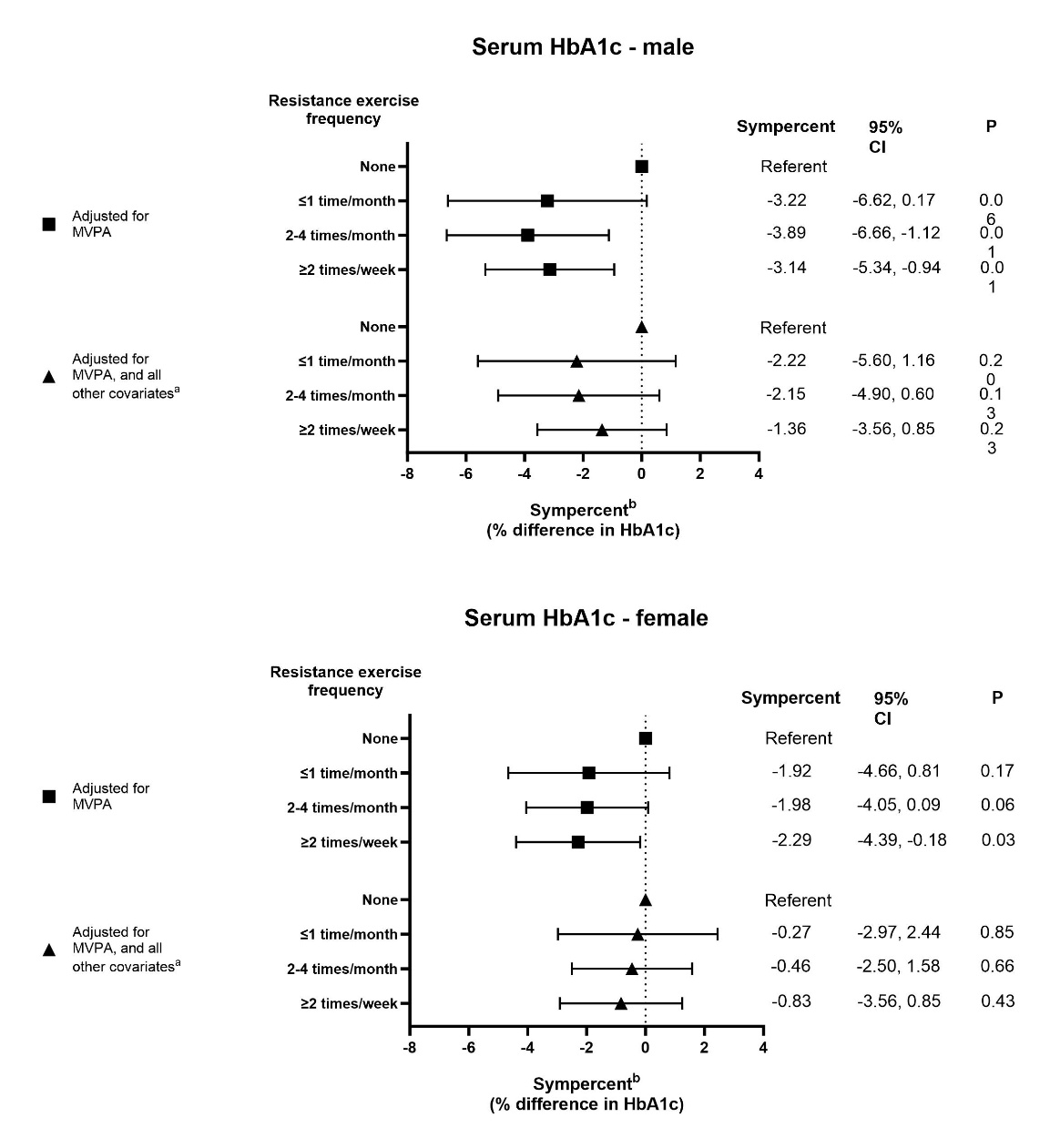


^a^Other covariates include smoking status, alcohol consumption, educational attainment, self-rated health, and disability

^b^Sympercent represents the percentage difference after transformation of the outcome variable into the 100 log_e_ scale

CI, confidence interval; HbA1c, haemoglobin A1c; MVPA, moderate-vigorous physical activity.
